# Silver nanoparticles versus chitosan nanoparticles effects on demineralized enamel

**DOI:** 10.1186/s12903-024-04982-4

**Published:** 2024-10-24

**Authors:** Mariam Aboayana, Marihan I. Elgayar, Mohamed H. A. Hussein

**Affiliations:** 1 Department of Oral Biology, Faculty of Dentistry, Alexandria University, Elmassalah, Alexandria, Egypt; 2 Department of Conservative Dentistry, Faculty of Dentistry, Alexandria University, Alexandria, Egypt

**Keywords:** Demineralization, Noncarious lesions, Enamel, Remineralization, Chitosan nanoparticles, Silver nanoparticles

## Abstract

**Background:**

To compare the impacts of different remineralizing agents on demineralized enamel, we focused on chitosan nanoparticles (ChiNPs) and silver nanoparticles (AgNPs).

**Methods:**

This study was conducted on 40 extracted human premolars with artificially induced demineralization using demineralizing solution. Prior to the beginning of the experimental procedures, the samples were preserved in artificial saliva solution. The nanoparticles were characterized by transmission electron microscopy (TEM) and teeth were divided into four equal groups: Group A was utilized as a control group (no demineralization) and received no treatment. Group B was subjected to demineralization with no treatment. Group C was subjected to demineralization and then treated with ChiNPs**.** Group D was subjected to demineralization and then treated with AgNPs. The teeth were evaluated for microhardness. The enamel surfaces of all the samples were analysed by scanning electron microscopy (SEM) for morphological changes and energy dispersive X-ray analysis (EDX) for elemental analysis.

**Results:**

The third and fourth groups had the highest mean microhardness and calcium (Ca) and phosphorous (P) contents. SEM of these two groups revealed relative restoration of homogenous remineralized enamel surface architecture with minimal micropores.

**Conclusion:**

Chitosan nanoparticles (NPs) and silver NPs help restore the enamel surface architecture and mineral content. Therefore, chitosan NPs and AgNPs would be beneficial for remineralizing enamel.

## Background

Recently, the focus of a carious inquiry has switched to the development of techniques for early disease diagnosis of caries, which will enable a non-invasive therapeutic procedure. The deposition of inorganic ions from the hydroxyapatite (HA) crystal complex is a process known as enamel demineralization. When the tooth surface is exposed to an acidic challenge, demineralization occurs [1]. In shallow enamel lesions, demineralization is thought to be a repairable process. Generally, if a tooth is subjected to oral conditions that encourage remineralization, its partially demineralized crystalline HA network may regenerate to its primordial form [2].

Remineralization is a process that helps to restore the lost minerals in the enamel and prevent further damage**.** Remineralization is a natural process of tooth healing in which P and Ca ions from plaque and salivary glands are deposited into crystal gaps in the demineralized tooth structure, producing a net mineral gain. Ca and P ions can be incorporated into the crystal lattice by free fluoride (F) ions in the oral environment. This results in fluorapatite minerals that are substantially more resistant to acid challenge [3, 4].

In order to minimize the side effects of extremely conservative surgical treatments and lessen the need for surgical intervention for lesions, non-restorative therapeutic methods have been emphasized as promising resources. This is accomplished by minimizing the loss of dental tissues by restoring their biological and physical characteristics through various remineralization techniques [5, 6].

Historically, the first attempt at remineralizing enamel was achieved with fluoride; remineralizing compounds have been studied to restore demineralized enamel, and biomimetic approaches have been proposed to re-establish enamel demineralization [7, 8]. Because of the amount of Ca and P ions available in the salivary fluid, there is limited remineralization even though compared to HA crystals, fluoride is more stable and resistant to acid [9, 10].

Then other techniques were used such as, the use of tricalcium phosphate, nano hydroxyapatite, probiotics, sodium bicarbonate, calcium hydroxide, lasers, stannous fluoride-containing toothpastes or gels, acidulated phosphate fluoride gels (APF), difluorsilane, ammonium fluoride, polyols, calcium phosphate, amorphous calcium phosphate (ACP), tricalcium phosphate, probiotics, and/or 1.5% arginine, probiotics, silver diammine fluoride (SDF), silver nitrate, lasers, and carbamide peroxide [6, 11–13].

Moreover, casein phosphopeptide–amorphous calcium phosphate (CPP-ACP)/casein phosphopeptide–amorphous calcium fluoro phosphate–amorphous calcium fluoro phosphate (CCP-ACFP were utilized. These soluble calcium-phosphate ions speed up remineralization and produce a form that is resistant to acid assaults. Furthermore, it has been observed that the combination of CPP-ACP and flouride (ACFP) has a synergistic impact on caries prevention) [14].

Depending on how closely the appearance and crystal structure of nanoparticles resemble those of enamel apatite crystals, various remineralizing agents in the form of nanoparticles, such as chitosan nanoparticles and silver nanoparticles, have been developed to aid in this process.

As a controlled medication delivery strategy, the ChiNPs suspension can be mixed with mouthwash. When cationic chitosan and anionic substances like sodium tripolyphosphate (TPP) are combined, the ionic gelation process produces hydrophobic precipitates (HNPs), which are gel-like nanoparticles. Anionic (P-O-) and cationic (NH3 +) groups interact through Lewis acid–base three-dimensional cross-links to stabilize these structures, which causes the chemical to precipitate from an aqueous solution [15, 16]. The regulated distribution of drugs through ionic dissociation during dilution is possible with this suspension of nanoparticles [17]. Furthermore, it exhibits mucoadhesion characteristics and creates a physical barrier from which the active ingredients can be gradually and precisely released into cheek, gingiva, alveolar mucosa of the oral epithelium and the enamel surface [18].

Chitosan nanoparticles have gained attention as potential remineralizing agents due to their ability to penetrate the enamel surface and promote mineral deposition. ChiNPs have been demonstrated in numerous studies to augment enamel remineralization and enhance the surface's mechanical characteristics [19, 20]. Furthermore, the antimicrobial characteristics of chitosan nanoparticles may aid in preventing further decay [21].

Silver nanoparticles have also been investigated as potential remineralizing agents due to their ability to inhibit bacterial growth and promote mineral deposition. Research has demonstrated that the mechanical characteristics of the enamel surface and enamel remineralization can both be enhanced by AgNPs [22]. Furthermore, it has been demonstrated that AgNPs possess antimicrobial qualities, which may aid in halting additional deterioration [23].

Furthermore, The spherical shape and small size of the NSF nanoparticles increase the contact surface, significantly enhancing the antibacterial capabilities [24, 25]. The antibacterial and cytotoxic properties of NSF, chlorhexidine, and SDF were compared in a study conducted by Targino et al. [26] NSF was discovered to be both bactericidal and bacteriostatic. It was determined that NSF was not hazardous at the employed quantities for any kind of red blood cell and was two to three times more biocompatible than SDF [27].

The null hypothesis, which was evaluated, was that the microhardness, histology, and composition of enamel did not significantly differ between the study groups that received nanoparticle treatment.

The purpose of this study was to compare the impacts of ChiNPs and AgNPs on demineralized enamel.

## Methods

### Study design (Invitro study)

The sample size was determined at the 95% confidence level to determine changes in the microhardness between the chitosan nanoparticles and silver nanoparticles. One study reported the mean standard deviation (SD) of the microhardness after remineralization using ChiNPs = 315.4 (20.2) [13]. Additionally, Favaro et al. reported a mean (SD) final microhardness of 155.4 (31.8) for AgNPs. The calculated mean (SD) difference was 160.0 (26.0), and the 95% confidence intervals were 135.71 and 184.29 [28]. The minimum sample size of nine teeth was determined; however, it was increased to ten to account for faults in laboratory processing. The total number of groups times the number of teeth in each group equals 40, or 4 × 10 [29].

### Setting

The public health department of the Alexandria University Faculty of Dentistry used version 19.0.5 of the software MedCalc Statistical Software (MedCalc Software bvba, Ostend, Belgium; https://www.medcalc.org; 2019).

#### Materials

- Extracted human teeth (total) *n = *40
- Chemicals as demineralizing agents
- Chitosan nanoparticles
- Silver nanoparticles

### Sample selection

Forty freshly non-carious extracted premolar teeth were utilized. The extracted sound premolar teeth were removed from the oral surgery department for orthodontic therapy. The soft tissues and calcified tissue on the teeth were manually removed before storage in 0.1% thymol until the beginning of the experiment.

### Sample demineralization preparation

Forty specimen surfaces were coated with dark nail polishing from FLORMAR COSMETICS purchased from Egyptian market, except that only a 4 mm-diameter window was left unprotected. The nail polished samples were submerged in a demineralizing solution (2.2 mM CaCl2, 10 mM NaH2PO4, 50 mM acetic acid, 100 mM NaCl, 1 ppm NaF, 5 mM NaN3; pH 4.5; degree of saturation respecting enamel 4.44 × 10 − 9) to produce lesions known as white spots. Before the experiment started, the samples were kept in artificial saliva solution (0.13 mM KCl, 1.5 mM CaCl2, 0.9 mM NaH2PO4, and 5 mM NaN3; pH 7.0) for only one day at 37 °C in an incubator [30].

#### Methodology

This study was applied to extracted human premolars with artificially induced demineralization. The nanoparticles were purchased from Nano Gate company. Chitosan nanoparticles were prepared according to the ionotropic gelation process. Blank nanoparticles were obtained upon the addition of a tripolyphosphate (TPP) aqueous solution to a Chitosan solution. Briefly, 1gm of Chitosan powder was dissolved in 200 ml 1% acetic acid (pH = 4) and stirred for 6 h to get homogenous solution then, add 150 ml of TPP 0.2% w/v dropwise. The clear solution turned to turbid indicating formation of CSNPs after that, the suspension was washed by centrifugation for 30 min at 15,000 rpm (Hermle Z32 HK, Germany) three times with DH_2_O [31–34].

Silver nanoparticles have been prepared by chemical reduction method as reported by Turkevich [35], Lee and Meisel [36]. A solution of AgNO_3_ (Merck, Germany) has been used as Ag^1+^ ions precursor. The PVP (Loba-India) has been used as stabilizing agent and sodium borohydride (Loba-India) as mild reducing agent. The color of the solution slowly turned into grayish yellow, indicating the reduction of the Ag^1+^ ions to Ag nanoparticles.

**Fig. 1:**
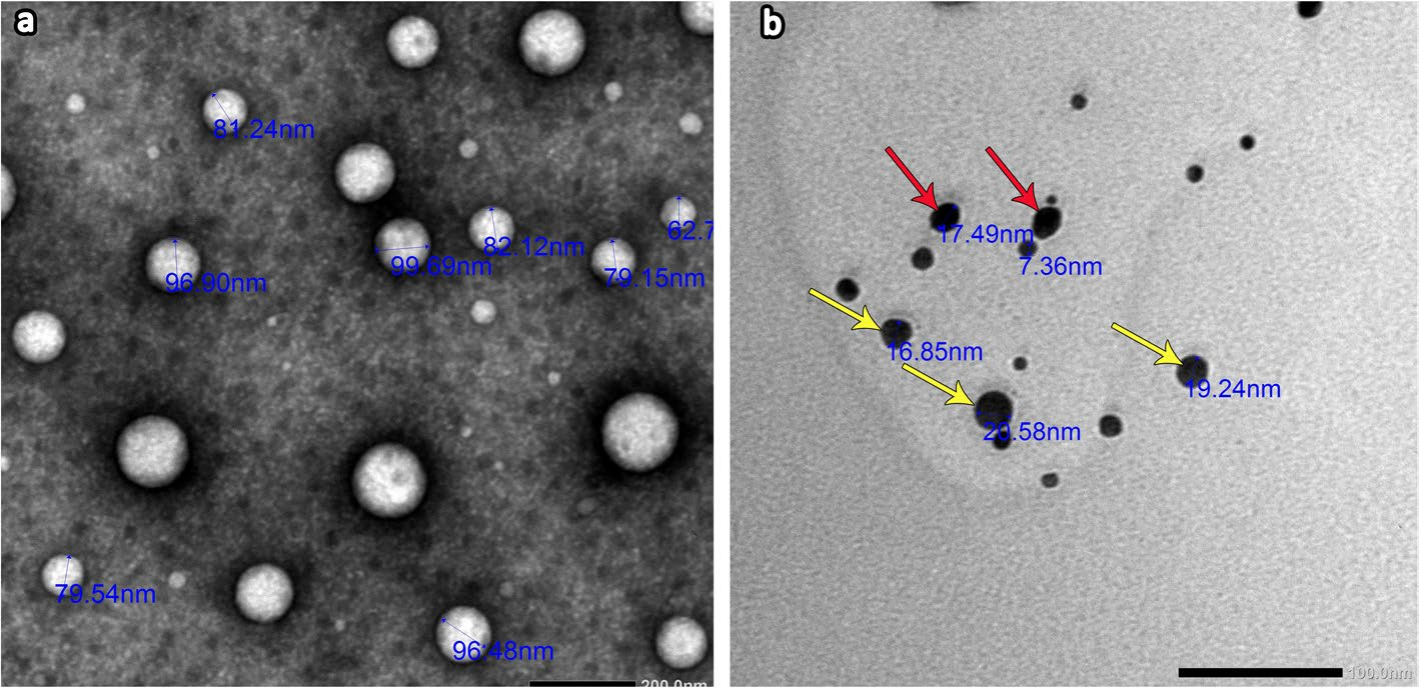
Transmission electron micrograph of a ChiNps suspension (**a**) showing nanoparticles with a typical spherical shape < 100 nm in diameter (2000x). The AgNP suspension (**b**) revealed spherical (yellow arrows) and oval-shaped (red arrows) nanoparticles with diameters < 30 nm (2000x)

The nanoparticles were characterized by transmission electron microscopy (Fig. 1). Figure 1a shows TEM images of ChiNPs with typical spherical shapes less than 100 nm in diameter. Figure 1b shows a TEM image of AgNPs with a diameter less than 30 nm. They mainly had a spherical shape (yellow arrows), while the others had an oval shape (red arrows).

#### After that, the teeth were split into four groups

- Group A was utilized as a control group (no demineralization) and received no treatment (control-ve) (*n = *10).
- Group B was subjected to demineralization with no treatment (control + ve) (*n = *10).
- Group C was subjected to demineralization then, the samples were washed by distilled water after that immersed in ChiNPs solution (*n = *10).
- GROUP D was subjected to demineralization then, the samples were washed by distilled water after that immersed in AgNPs solution (*n = *10).

#### Preparation for assessment

The specimens were rinsed with a water spray and then gently polished with polishing paste. Subsequently, an ultrasonicator was used in a water bath for clearance of the enamel surface from any residue of the remineralizing agents and stored in artificial saliva solution.

#### Assessment

**Fig. 2:**
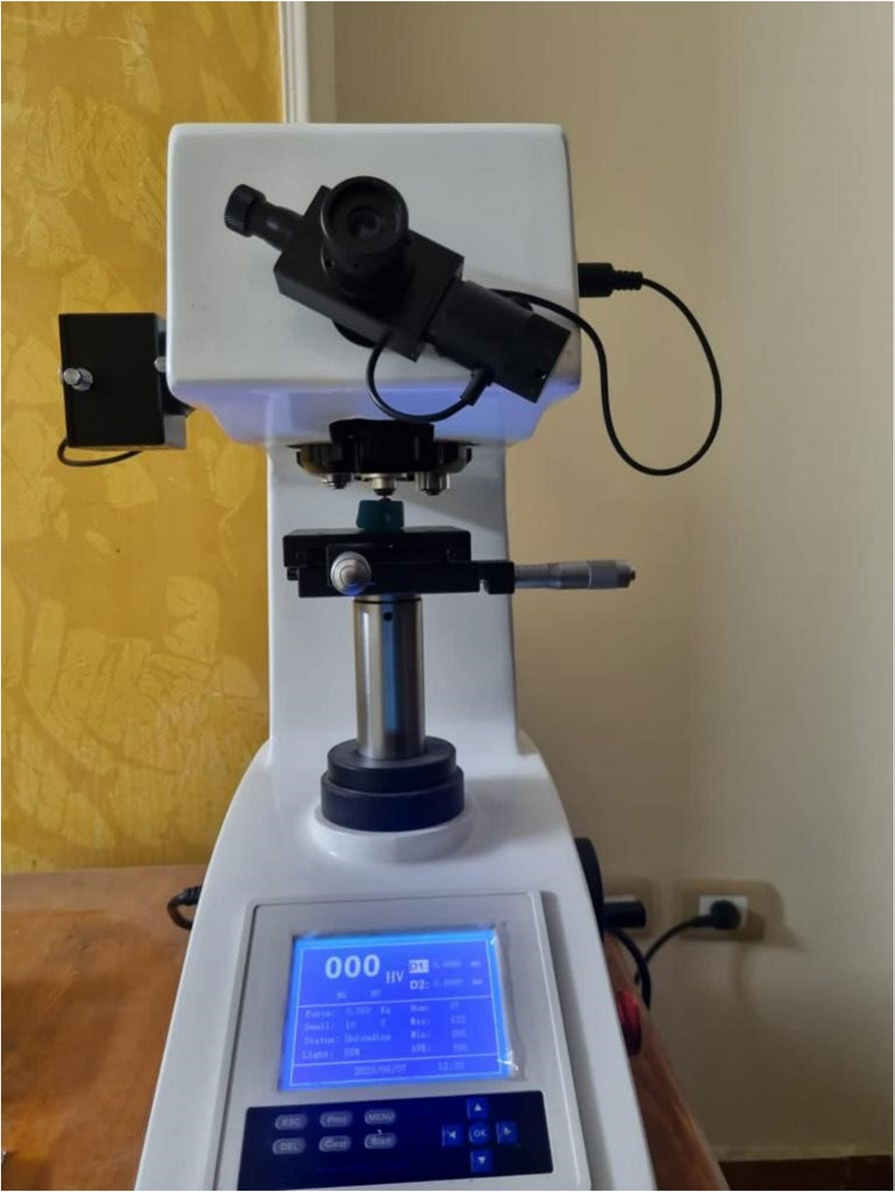
Digital autoturret micro-Vickers hardness tester

- 1. *Microhardness analysis*: All teeth of each group (*n = *10) were evaluated for microhardness by the Vickers hardness test (VHN) 50 gm every 10 s after one day from time of application of remineralizing agents to treated groups (Fig. 2). Three indentations at a distance of 100 μm each were made in each sample for each group, and the mean values of the three indentations indicated the sample's value in the baseline [37].
- 2. *Morphological analysis*: Ten specimens from each group (*n = *10) were SEM-examined after one week from time of application of remineralizing agents to treated groups. The specimens were carefully dried and gold-coated using the JFC-1100E-IEOL ion sputtering evaporator. After that, the specimens were put on a SEM (JSM-IT200, JEOL) to examine the morphology of the enamel surface [38].
- 3. *Elemental analysis and chemical characterization*: The enamel surface for all the samples of each group (*n = *10) was analysed using EDX for elemental analysis. The calcium, phosphate and carbon contents of surface enamel was measured for each specimen with their percentages. This was achieved with EDX. Data were recorded within an average of 6 min for each specimen immediately before gold coating of SEM specimens [13, 38].
- 4. *Statistical analysis*: Descriptive statistics, normality tests, and plots (boxplots, Q‒Q plots, and histograms) were used to verify normality. Since all of the data had a normal distribution, parametric tests were performed, and the means and SDs were calculated. One-way ANOVA was used to compare the four research groups, and repeated pairwise comparisons with a Bonferroni adjusted significance level were then conducted. A *p* value < 0.05 indicated statistical significance. Version 26.0 of IBM SPSS for Windows was used to analyse the data.

## Results

### Surface microhardness test results

**Fig. 3:**
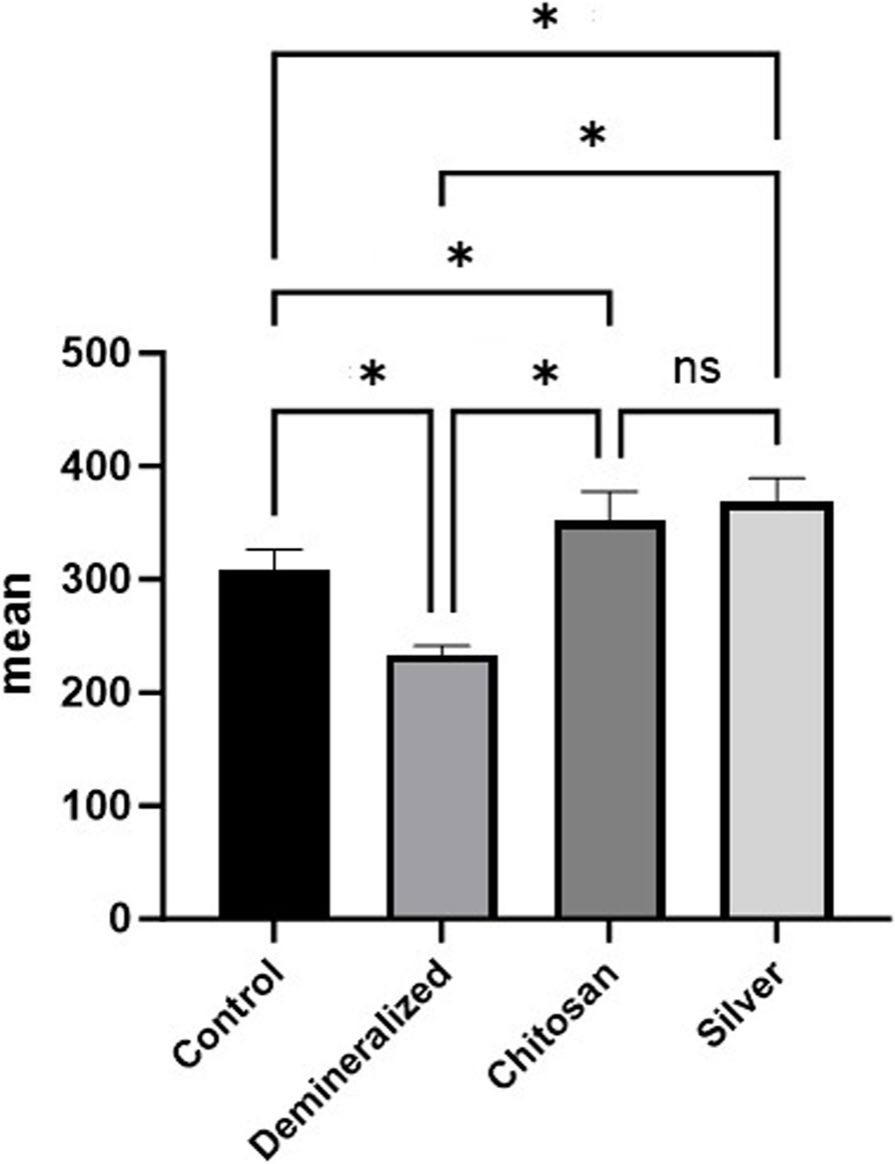
Simple bar chart showing comparisons between the different groups according to the percentage change in the MH

Table 1 and Fig. 3 show comparisons between the VHN of the four study groups. The last group, which was subjected to demineralization and then treated with AgNPs, showed the highest mean value compared to the other groups. On the other hand, the second group that was subjected to demineralization with no treatment had the lowest mean value. Post hoc comparisons between the four study groups revealed statistically significant differences according to the Bonferroni correction (*P* value <0.001*); however, the differences between the third and fourth study groups were not statistically significant (*P* value = 0.44).

**Table 1:** Comparison of microhardness among the four groups (*n = *10)

Group	Mean (SD)	Median (IQR)	Min – Max
**Control**	307.90 (18.41)^**a**^	300.50 (33.50)	289.00 – 340.00
**Demineralized**	233.70 (7.63)^**b**^	232.50 (14.75)	224.00 – 246.00
**ChiNPs**	351.90 (2.83)^**c**^	353.00 (34.50)	299.00 – 390.00
**AgNPs**	368.00 (21.40)^**c**^	373.50 (32.25)	328.00 – 396.00
***P* value**	< 0.001^*^		
**Post hoc comparisons** **(*p* value)**	Control vs. Demineralized: < 0.001^*^		
	Control vs. Chitosan: < 0.001^*^		
	Control vs. Silver: < 0.001^*^		
	Demineralized vs. Chitosan: < 0.001^*^		
	Demineralized vs. Silver: < 0.001^*^		
	**Chitosan vs. Silver:** 0.44		

*SD* standard deviation, *IQR* interquartile range, *Min* minimum, *Max* maximum

^*^Statistically significant at *p* value < 0.05

^a^^−^^c^The letters indicate statistically significant differences between groups after Bonferroni adjustment

### SEM results

#### Group 1 (Control group)

**Fig. 4:**
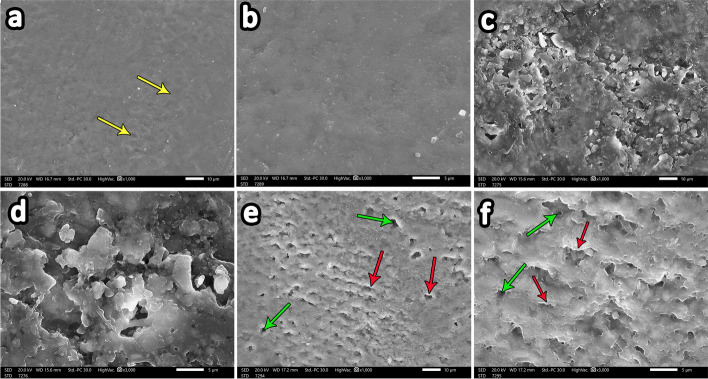
Scanning electron microscopy (SEM) image of control group specimens (**a**) showing a homogenous enamel surface with a typical fish scale appearance of enamel rods (yellow arrows) (magnification X1000). Higher magnification of the previous image of the control group specimen (**b**) showing a smooth prismless layer free from porosity (magnification X3000). SEM of the demineralized group (**c** and **e**) revealed a marked erosive pattern with a rough enamel surface and loss of the prism core (green arrows) while retaining the periphery (red arrows). X1000. Higher magnification of the previous images showing damage to the prismatic pattern (**d**) with destruction of the prism core (green arrows) but keeping the periphery intact (red arrows). **f** (magnification, X3000)

The entire enamel specimen showed a typical fish scale with enamel rods (yellow arrows) and a regular smooth surface architecture. Higher magnification showed a homogenous prismless layer free from porosity or erosion covering most of its surface (Fig. 4a and b).

#### Group 2 (Demineralization group)

The examination revealed a marked erosive pattern with a rough enamel surface. At higher magnification, excessive porosities were evident (Fig. 4c and d).

Disorganized and irregular enamel surfaces with multiple porosities were significant. Damage to the prismatic pattern was noticeable at higher magnification with destruction of the prism core (green arrows) but keeping the periphery intact (red arrows) (Fig. 4e and f).

#### Group 3 (Demineralization then ChiNPs)

**Fig. 5:**
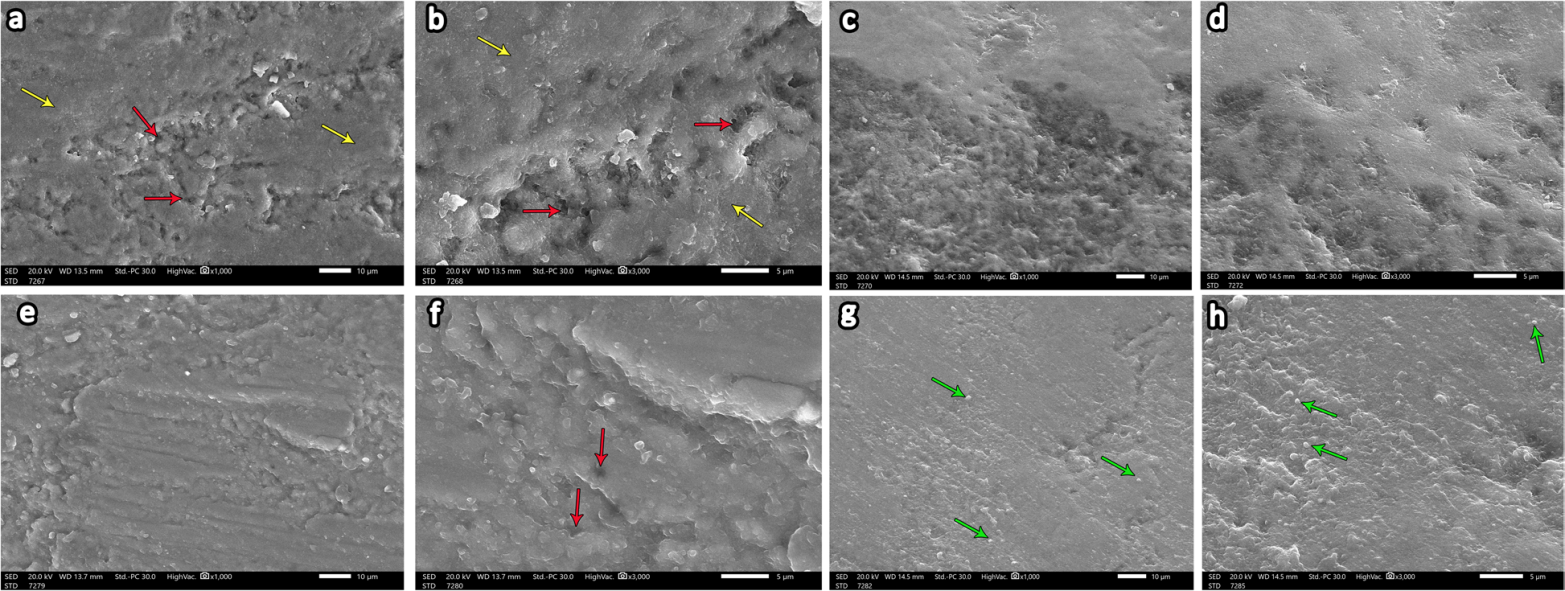
SEM of the ChiNP group specimens (**a**-**d**) revealed a relatively homogenous enamel surface with a reduction in porosity and a less distinct erosive pattern (**a** and **c**) (X1000). At higher magnification, demineralization is less obvious, with minimal loss of the prism core. **b** and **d** (X3000). Some areas with microporosities (red arrows) alternating with other smooth areas (yellow arrows) (**a** and **b**). SEM of the AgNP group specimens (**e**-**h**) revealed that the smooth enamel architecture (**e**) was almost completely restored, and a dense coating on the remineralization surface was observed, with white spots indicating silver nanoparticles on the sample (green arrows) (**g**) (X1000). Higher magnification revealed minimal micropores (red arrows) (**f**) with spotting of mineral particles on the enamel surface (green arrows), creating a layer of remineralized crystals (**h**) (X3000)

Meticulous features were revealed on the relatively homogenous enamel surface with a reduction in porosity. Higher magnification revealed some areas with microporosities (red arrows) alternating with other smooth areas (yellow arrows) (Fig. 5a and b).

The specimens exhibited a less distinct erosive pattern and were less aggressive than those in group 2. At higher magnification, demineralization was less obvious, with minimal loss of the prism core (Fig. 5c and d).

#### Group 4 (Demineralization then AgNPs)

In this group, demineralization was less distinct than that in group 2, with almost complete restoration of the homogenous enamel surface architecture (Fig. 5e). Minimal micropores were observed at higher magnification (red arrows). (Fig. 5f).

A dense coating on the remineralization surface was observed with white spots, indicating that silver nanoparticles were present throughout the sample (green arrows). Higher magnification revealed the spotting of mineral particles on the enamel surface (green arrows), creating a layer of remineralized crystals (Fig. 5g and h).

### EDX

**Fig. 6:**
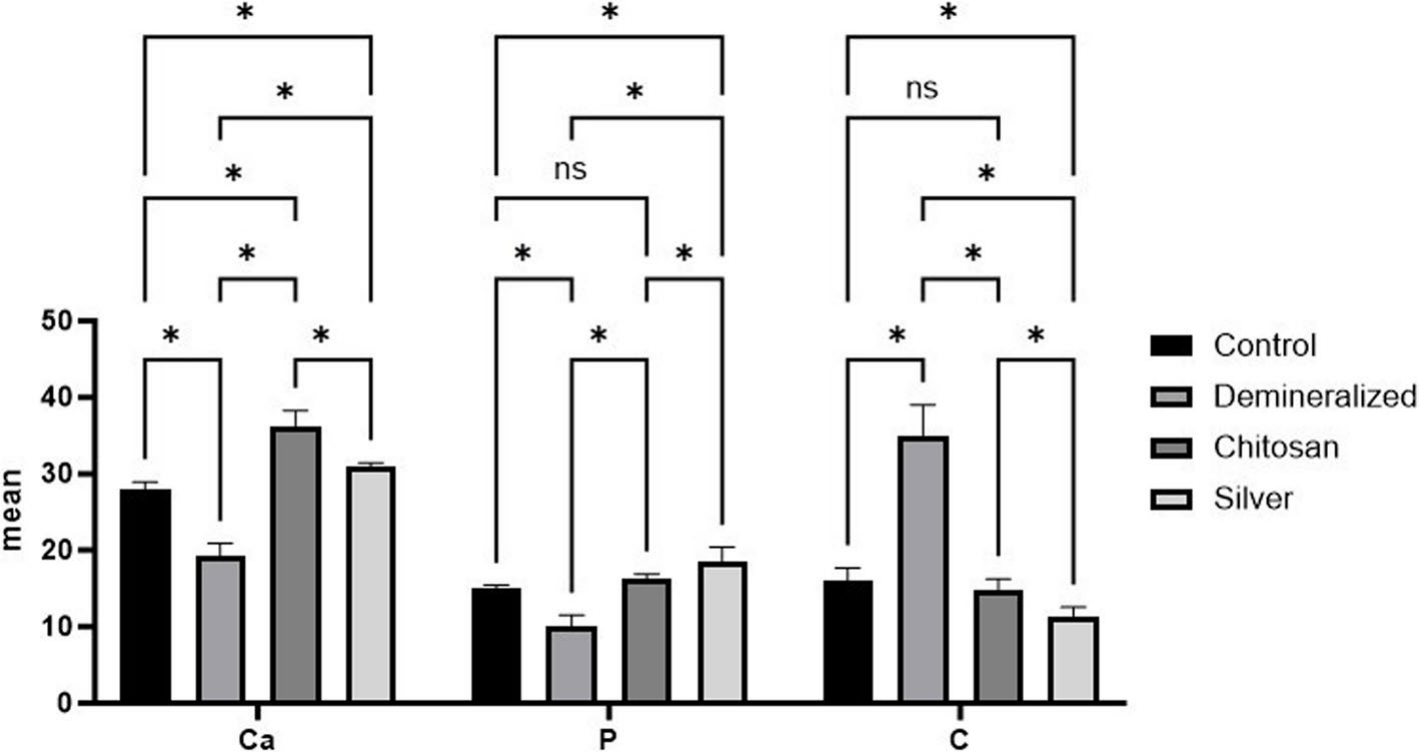
Bar chart revealing comparisons between the different groups according to the percentages of Ca, P and C

**Fig. 7:**
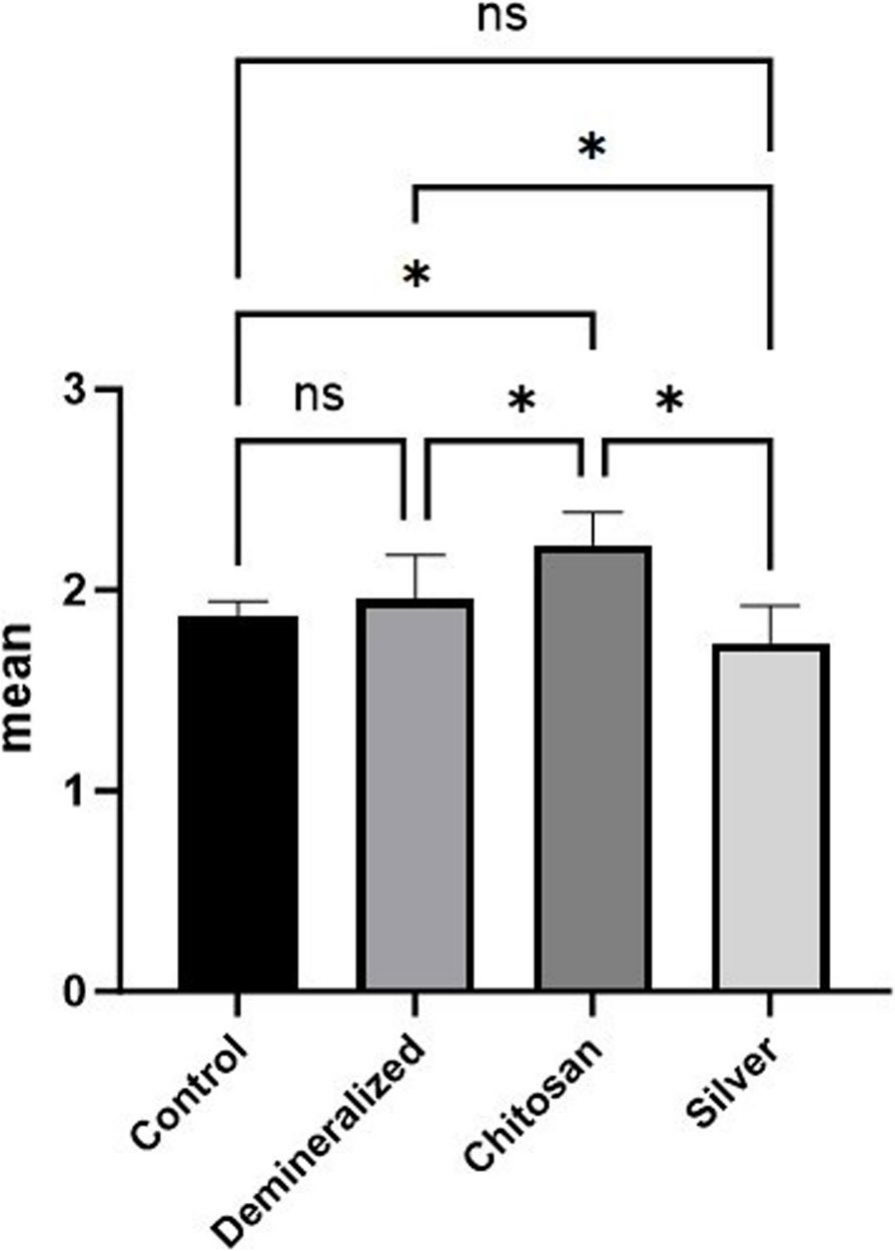
Simple bar chart demonstrating comparisons between the different groups regarding the Ca/P ratio

According to the statistical results shown in Tables 2 and 3 and Fig. 6, the highest Ca mass% and Ca/P ratio (Fig. 7) were detected in the ChiNP group (gp3), while the highest P and lowest C mass% were detected in the AgNP group (gp4). In contrast, the highest C mass % with the lowest Ca and P mass % were apparent in demineralization group 2. Among the groups, group 3, which had the highest Ca mass percentage, exhibited a significant difference from group 4 (*P* < 0.001).

**Table 2:** Comparisons of the EDX results between the four groups. *(n = 10)*

	**Control**	**Demineralized**	**ChiNPs**	**AgNPs**	***P* value**
	**Mean (SD)**				
**Ca**	27.96 (0.99)^**a**^	19.27 (1.61)^**b**^	36.26 (2.00)^**c**^	30.87 (0.55)^**d**^	< 0.001^*^
**P**	14.98 (0.44)^**a**^	10.02 (1.52)^**b**^	16.29 (0.59)^**a**^	17.98 (2.12)^**c**^	< 0.001^*^
**Ca:P**	1.87 (0.08)^**ac**^	1.95 (0.22)^**a**^	2.23 (0.16)^**b**^	1.74 (0.19)^**c**^	< 0.001^*^
**C**	16.01 (1.69)^**a**^	34.95 (4.08)^**b**^	14.79 (1.50)^**a**^	11.43 (1.12)^**c**^	< 0.001^*^

*SD* Standard deviation

^*^Statistically significant at *p* value < 0.05

^a^^−^^c^Different letters indicate statistically significant differences between groups utilizing Bonferroni adjustment

**Table 3:** Post hoc comparisons of the EDX results among the four groups

	Group	Compared to	*P* value
**Ca**	**Control**	**Demineralized**	< 0.001^*^
		**ChiNPs**	< 0.001^*^
		**AgNPs**	< 0.001^*^
	**Demineralized**	**ChiNPs**	< 0.001^*^
		**AgNPs**	< 0.001^*^
	ChiNPs	**AgNPs**	< 0.001^*^
**P**	**Control**	**Demineralized**	< 0.001^*^
		**ChiNPs**	0.22
		**AgNPs**	< 0.001^*^
	**Demineralized**	**ChiNPs**	< 0.001^*^
		**AgNPs**	< 0.001^*^
	**Chitosan**	**AgNPs**	0.04^*^
**Ca:P**	**Control**	**Demineralized**	1.00
		**ChiNPs**	< 0.001^*^
		**AgNPs**	0.57
	**Demineralized**	**ChiNPs**	0.005^*^
		**AgNPs**	0.04^*^
	**Chitosan**	**AgNPs**	< 0.001^*^
**C**	**Control**	**Demineralized**	< 0.001^*^
		**ChiNPs**	1.00
		**AgNPs**	0.001^*^
	**Demineralized**	**ChiNPs**	< 0.001^*^
		**AgNPs**	< 0.001^*^
	**Chitosan**	**AgNPs**	0.02^*^

^*^Statistically significant at the Bonferroni-adjusted significance level

There was a statistically significant difference in P mass % between group 4 and the other three groups (*p* < 0.05). However, the *P* values in groups 1 and 3 were not significantly different (*p* > 0.05).

The C mass % mean was significantly greater in group 2 than in groups 1, 3 and 4 (*P* < 0.001).

Concerning the Ca/P ratio (Fig. 6B), there was a significant difference between gp3, which had the highest value, and the other gps (*p* < 0.05). Nevertheless, no significant difference was observed between the control gp1 and either group 2 or group 3 (*p* > 0.05).

## Discussion

Enamel is an exclusively epithelial-derived dental tissue that possesses a unique microstructure and outstanding physicochemical properties. To combat the challenge of cariogenic diseases that cause enamel demineralization, a number of approaches have been applied [26]. The aim of these approaches is the restoration of demineralized enamel due to the unrestorable nature of tooth enamel by different remineralizing agents [39].

The current in vitro research aimed to compare the effects of two different remineralizing agents, ChiNPs and AgNPs, on demineralized enamel.

In the present study, chitosan nanoparticles characterization by transmission electron microscope revealed that the individual particles were typical spherical in shape with a diameter less than 100 nm. This result was in harmony with El-Naggar et al. as their TEM images of ChiNPs indicated their spherical morphology but the size was less than 20 nm [40]. Khanmohammadi et al who utilize field emmission-SEM, observed that the synthesized ChiNPs had particle sizes with an average between 33 -74 nm [41].

Moreover, TEM analysis of the AgNPs revealed that the nanoparticles were mainly spherical while others oval with their diameter less than 30 nm. This is in accordance with Favaro et al. who observed AgNPs predominantly spherical in shape with sizes ranging from 7–30 nm [28].

Microhardness test, SEM and EDX were applied in the current study for monitoring enamel changes between the four groups. The determination of the mineral content on the surface of the enamel using EDX had been used to measure mineral loss and uptake [42]. In conjunction with SEM, energy dispersive X-ray analysis (EDX) is carried out. It offers the general spatial mapping inside a sample as well as the elemental specifics of the near-surface components. Moreover, it creates a specimen map to examine the types and quantities of elements at the nanomaterial surface or in the vicinity of the surface [43]. The atomic structure of the element from which the X-rays originate determines the energy of the X-rays produced, which in turn gives the elemental specifics of the sample. Since X-rays are produced at a depth of around 2 μm in the material, EDX is often used for bulk characterization. To confirm the sample's spatial homogeneity and uniformity, the electron beam is scanned across it [44].

The hardness of remineralized surfaces had been assessed using a VHN. It is one of the most traditional methods for determining hardness, and it works with most metals and welds thanks to its broad hardness scale. Compared to other hardness tests, the Vickers hardness test is frequently seen to be simpler to use: The steps can be carried out using a universal or micro hardness tester; the calculations needed don't depend on the indenter's size; and all materials—hard or soft—can be utilized with the same indenter, a pyramidal diamond [42, 45].

A scanning electron microscope is a scientific device that produces high-resolution images by using a concentrated electron beam to study a specimen's three-dimensional structure and minute surface features. It fills the void left by the better resolving power of the transmission electron microscope and the magnification powers of light microscopy [46]. SEM detected ultrastructural changes on the surface of teeth with morphologic topographies of remineralization effects [13].

When assessing the demineralized dental enamel's surface microhardness versus those treated with ChiNPs and AgNPs, a larger percentage of modification in superficial microhardness was confirmed on the surfaces managed with nanoparticles. De Carvalho et al explained that Chitosan uses the NH3 groups' electrostatic charges to draw phosphate ions from saliva. Additionally, the functional carboxyl found on chitosan surfaces causes efficient spontaneous apatite nucleation, which raises hardness values upon remineralization [47].

Furthermore, the study's findings concur with those of Yihong et al. and Noaman et al., who concurred that chitosan and silver diamine flouride (SDF) are more effective remineralization agents than NaF varnish [48, 49].

Additionally, the remineralization achieved in this research coincides with the study of Scarpelli et al. and Nozari A et al. who assessed the surface and depth remineralizing effects of an experimental solution containing AgNPs on human deciduous enamel. The performance of the experimental solution in terms of remineralizing was found to be inferior compared to SDF. Nevertheless, the authors noted that due to the challenge of stabilizing nanoparticles in interaction with fluoride, their solution contained just silver nanoparticles and no fluoride. Increased tooth enamel microhardness could arise from the precipitation of a silver ion solution that infiltrates carious lesions [50, 51].

Changes in enamel microhardness, as detected by a microhardness test, can be related to the mineral content of tooth structure. The antibacterial activity of silver ions is attributed to their reaction with hydroxyapatite crystals, which precipitates silver phosphate and produces an insoluble, hard coating on the surface of enamel. Furthermore, phosphate ions from saliva are drawn to chitosan by the NH3 groups' electrostatic forces. Effective spontaneous apatite nucleation occurs in an ordered direction throughout the surface of chitosan due to the presence of a functional carboxyl on its surfaces. This might explain the hardness test results that indicated increase in hardness values after remineralization to the extent of reaching high value of hardness [13].

Conversely, the result of this study did not coincide with the results observed in previous studies which demonstrated how well an experimental fluoride solution including AgNPs can prevent dental enamel caries and suggested that the combination of the formulation's ingredients could account for this result. The remineralization of dental enamel is thus caused by both F and AgNPs, whose actions can be amplified when combined [52–54].

Concerning scanning electron microscopic examination of the present study, the findings of the control group with sound teeth revealed smooth surface topography of the enamel surface without any porosity. Moreover, typical fish scale appearance representing the shape of enamel rods was clearly identified. Soares et al. confirmed that the sound enamel revealed enamel rods in well-organized pattern with homogeneously arranged enamel crystals [55].

Upon monitoring data attained from the current study regarding the demineralizing group, it revealed rough and disorganized enamel surface with excessive erosions. It also exhibited damage of the prismatic pattern with destruction of prism core but keeping the periphery intact. These results were in accordance with Wang et al. who observed dis-ordered arrangement of the enamel rods with losing enamel rods core however, the periphery was retained. Furthermore, Alvati et al. confirmed rough enamel surface with irregular pitting and damage of the prism pattern [56, 57].

Other findings by Besnard et al. who discovered variant forms of enamel defects such as, damage of enamel rod core, destruction of rod periphery whereas rod core persisted intact [58].

The results obtained from the ChiNP group in the present study showed alternative layers of relatively homogenous areas on the enamel surface, with other areas exhibiting microporosities. Demineralization was less obvious with minimal loss of the prism core, indicating an enamel response to remineralization.

Recent research revealed a comparatively smooth enamel surface in the phosphorylated ChiNP group, in which some minerals precipitated. Some widening of the interprismatic distance was observed due to the loss of enamel rod peripheries, while the rod cores remained intact. Nonetheless, some areas exhibited microporosities due to the loss of prism cores, whereas peripheries were intact [13].

The results of the silver nanoparticle group in the present study revealed the relative restoration of homogenous enamel surface architecture with minimal micropores. Additionally, a dense coating of the remineralization surface with white spots was observed representing silver nanoparticles that precipitated over the sample, creating a layer of remineralized crystals.

These results are in agreement with those of Mohammed in 2022, who reported that the enamel surface of silver diamine fluoride-treated plants was relatively intact and dense compared to those of plants in other groups, with a decrease in the porosity of the smooth enamel surface. Moreover, white spots indicating silver were spotted over a dense layer of remineralization surface [13]. Furthermore, Favaro et al. observed that a layer of precipitate condensed with clustered AgNPs over the enamel surface [28].

Alsherif et al. showed few demineralized areas alternating with homogenous areas, and calcium deposits were detected sealing the enamel surface with spherical precipitates [38].

According to the EDX results of the present study, the percentages of Ca and P were greater in the ChiNP group and silver nanoparticle group than in the other groups. However, the C content in these two groups was lower than that in demineralization group II.

These results were in agreement with research in 2023, which revealed a marked decrease in the percentage of C and an increase in the percentage of Ca in the chitosan and silver groups in comparison to those in the group subjected to demineralization with no treatment. However, the percentage of P in the phosphorylated chitosan group was greater than that in the SDF group [13].

Additionally, the findings of Mei et al. and Li et al. agreed with the results of the present study, which showed that SDF’s mode of action was based on the reaction of silver with the P found in the demineralized lesion to generate silver phosphate that covers the enamel surface in an insoluble, hard coating [49, 59].

Furthermore, phosphate ions from saliva are drawn to chitosan by electrostatic interactions between the NH3 groups. Additionally, the efficient spontaneous nucleation of apatite in an ordered orientation across the surface of chitosan is caused by the functional carboxyl that is located on its surface [47].

Regarding the Ca/P ratio in the current study, the chitosan nanoparticle group had the highest value, which was significantly different from that of the other groups. However, no significant difference was observed between the control gp1 and either the demineralization group or the silver nanoparticle group. These results agreed with those of Mohammed et al., who reported that the percentages of Ca and P in the chitosan group were greater than those in other groups, including the SDF group; however, no significant differences were detected between them [13].

Moreover, Zhi et al. and Alsherif et al. noted that nanosilver fluoride (NSF) sections had higher Ca/P ratios than did other untreated sections of the same sample, suggesting a slow rate of surface decalcification of enamel. Because silver ions may penetrate carious lesions, they contribute to the remineralization of enamel [38, 60].

Furthermore, the antibacterial and cytotoxic action of NSF against *Streptococcus mutans* was also demonstrated by Targino et al. [26] Other studies suggested that NSF possessed antibacterial qualities that might be prevented, and it was depicted as a potent agent that prevented cavities [61, 62].

*The limitations of the research* were mostly caused by time constraints, as monitoring remineralization capacity over an extended period would have produced more conclusive findings about the potential for remineralization of each procedure. Furthermore, remineralizing agents would have a more noticeable effect if they were used more than three times. Insufficient research on ChiNPs has made it difficult to evaluate the effectiveness of ChiNPs against other remineralizing agents. This study was conducted using extracted teeth, and additional in vivo research might improve the findings.

## Conclusions

Considering the limitations of the present study, the following conclusions can be drawn: 1- ChiNPs and AgNPs are beneficial for enamel remineralization. 2- ChiNPs and AgNPs help form enamel patterns that mimic the prismatic orientation of enamel rods, which aids in enamel tissue regeneration. 3- Further research should be conducted to corroborate the results of this study.

## Data Availability

All datasets and materials used and/or analysed during the current study are included in this published article.

## Abbreviations

Ca: Calcium
ChiNPs: Chitosan nanoparticles
EDXA: Energy dispersive X-ray analysis
F: Fluoride
HA: Hydroxyapatite
IQR: Interquartile range
Max: Maximum
Min: Minimum
NSF: Nano silver fluoride
P: Phosphorous
SEM: Scanning electron microscopy
SDF: Silver diamine fluoride
AgNPs: Silver nanoparticles
SD: Standard deviation
TEM: Transmission electron microscopy
VHN: Vickers hardness test

## Data availability

No datasets were generated or analysed during the current study.

## References

[1] Cummins D. The development and validation of a new technology, based upon 1.5% arginine, an insoluble calcium compound and fluoride, for everyday use in the prevention and treatment of dental caries. J Dent. 2013;41:S1–11. 10.1016/j.jdent.2010.04.002. 23985433 10.1016/j.jdent.2010.04.002

[2] Kim MJ, Lee MJ, Kim KM, Yang SY, Seo JY, Choi SH, Kwon JS. Enamel demineralization resistance and remineralization by various fluoride-releasing dental restorative materials. Materials (Basel). 2021;14:4554. 10.3390/ma14164554. 34443077 10.3390/ma14164554 PMC8402149

[3] Marthaler TM. Changes in dental caries 1953–2003. Caries Res. 2004;38:173–81. 10.1159/000077752. 15153686 10.1159/000077752

[4] Khare K, Bhusari P, Soni A, Malagi SK, Abraham D, Johnson L. A comparative evaluation of efficacy of biomin F and propolis containing toothpastes on dentinal tubule occlusion with and without use of an adjunct 810 nm diode laser: An In vitro scanning electron microscope study. J Microsc Ultrastruct. 2023;11:41. 10.4103/jmau.jmau_133_20. 37144169 10.4103/jmau.jmau_133_20 PMC10153734

[5] Ismail AI, Tellez M, Pitts NB, Ekstrand KR, Ricketts D, Longbottom C, et al. Caries management pathways preserve dental tissues and promote oral health. Community Dent Oral Epidemiol. 2013;41:e12-40. 10.1111/cdoe.12024. 24916676 10.1111/cdoe.12024

[6] Urquhart O, Tampi MP, Pilcher L, Slayton RL, Araujo MWB, Fontana M, et al. Nonrestorative treatments for caries: systematic review and network meta-analysis. J Dent Res. 2019;98:14–26. 10.1177/0022034518800014. 30290130 10.1177/0022034518800014 PMC6304695

[7] Amaechi BT, AbdulAzees PA, Alshareif DO, Shehata MA, Lima PPdCS, Abdollahi A, et al. Comparative efficacy of a hydroxyapatite and a fluoride toothpaste for prevention and remineralization of dental caries in children. BDJ Open. 2019;5:18. 10.1038/s41405-019-0026-8. 31839988 10.1038/s41405-019-0026-8 PMC6901576

[8] Zampetti P, Scribante A. Historical and bibliometric notes on the use of fluoride in caries prevention. Eur J Paediatr Dent. 2020;21:148–52. 10.23804/ejpd.2020.21.02.10. 32567947 10.23804/ejpd.2020.21.02.10

[9] Reynolds EC. Calcium phosphate-based remineralization systems: scientific evidence? Aust Dent J. 2008;53:268–73. 10.23804/ejpd.2020.21.02.10. 18782374 10.1111/j.1834-7819.2008.00061.x

[10] Hicks J, Garcia-Godoy F, Flaitz C. Biological factors in dental caries: role of remineralization and fluoride in the dynamic process of demineralization and remineralization (part 3). J Clin Pediatr Dent. 2004;28:203–14. 10.17796/jcpd.28.3.w0610427l746j34n. 15163148 10.17796/jcpd.28.3.w0610427l746j34n

[11] Karlinsey RL, Mackey AC, Walker ER, Frederick KE, Fowler CX. In vitro evaluation of eroded enamel treated with fluoride and a prospective tricalcium phosphate agent. J Dent Oral Hyg. 2009;1:52–8. http://www.academicjournals.org/jdoh.

[12] Juntavee A, Juntavee N, Sinagpulo AN. Nano-hydroxyapatite gel and its effects on remineralization of artificial carious lesions. Int J Dent. 2021;2021:7256056. 10.1155/2021/7256056. 34790238 10.1155/2021/7256056 PMC8592696

[13] Mohamed Y, Ashraf R. Remineralization potential of phosphorylated chitosan and silver diamine fluoride in comparison to sodium fluoride varnish: invitro study. Eur Arch Paediatr Dent. 2023;24:327–34. 10.1007/s40368-023-00794-2. 37014591 10.1007/s40368-023-00794-2 PMC10317901

[14] Aras A, Celenk S, Dogan MS, Bardakci E. Comparative evaluation of combined remineralization agents on demineralized tooth surface. Niger J Clin Pract. 2019;22:1546–52. 10.4103/njcp.njcp_188_19. 31719276 10.4103/njcp.njcp_188_19

[15] de Carvalho FG, Magalhães TC, Teixeira NM, Gondim BLC, Carlo HL, Dos Santos RL, et al. Synthesis and characterization of TPP/chitosan nanoparticles: Colloidal mechanism of reaction and antifungal effect on C. albicans biofilm formation. Mater Sci Eng C Mater Biol Appl. 2019;104:109885. 10.1016/j.msec.2019.109885. 31500048 10.1016/j.msec.2019.109885

[16] Zhang J, Boyes V, Festy F, Lynch RJM, Watson TF, Banerjee A. In-vitro subsurface remineralisation of artificial enamel white spot lesions pre-treated with chitosan. Dent Mater. 2018;34:1154–67. 10.1016/j.dental.2018.04.010. 29752161 10.1016/j.dental.2018.04.010

[17] Zhang J, Lynch RJM, Watson TF, Banerjee A. Chitosan-bioglass complexes promote subsurface remineralisation of incipient human carious enamel lesions. J Dent. 2019;84:67–75. 10.1016/j.jdent.2019.03.006. 30951785 10.1016/j.jdent.2019.03.006

[18] Magalhães TC, Teixeira NM, França RS, Denadai ÂML, Santos RLd, Carlo HL, et al. Synthesis of a chitosan nanoparticle suspension and its protective effects against enamel demineralization after an in vitro cariogenic challenge. J Appl Oral Sci. 2021;29:e20210120. 10.1590/1678-7757-2021-0120. 34644779 10.1590/1678-7757-2021-0120 PMC8523102

[19] Li D, Fu D, Kang H, Rong G, Jin Z, Wang X, Zhao K. Advances and potential applications of chitosan nanoparticles as a delivery carrier for the mucosal immunity of vaccine. Curr Drug Deliv. 2017;14:27–35. 10.2174/1567201813666160804121123. 27494157 10.2174/1567201813666160804121123

[20] Qu S, Ma X, Yu S, Wang R. Chitosan as a biomaterial for the prevention and treatment of dental caries: antibacterial effect, biomimetic mineralization, and drug delivery. Front Bioeng Biotechnol. 2023;11. 10.3389/fbioe.2023.1234758. 10.3389/fbioe.2023.1234758 PMC10570529 37840659

[21] Chandrasekaran M, Kim KD, Chun SC. Antibacterial activity of chitosan nanoparticles: a review. Processes. 2020;8:1173. 10.3390/pr8091173.

[22] Yun Z, Qin D, Wei F, Xiaobing L. Application of antibacterial nanoparticles in orthodontic materials. Nanotechnol. 2022;11:2433–50. 10.1515/ntrev-2022-0137.

[23] Yin IX, Zhang J, Zhao IS, Mei ML, Li Q, Chu CH. The antibacterial mechanism of silver nanoparticles and its application in dentistry. Int J Nanomed. 2020:2555–62. 10.2147/IJN.S246764. 10.2147/IJN.S246764 PMC7174845 32368040

[24] Agnihotri S, Mukherji S, Mukherji S. Size-controlled silver nanoparticles synthesized over the range 5–100 nm using the same protocol and their antibacterial efficacy. Rsc Advances. 2014;4:3974–83. 10.1039/C3RA44507K.

[25] Martínez-Castañon G-A, Nino-Martinez N, Martinez-Gutierrez F, Martínez-Mendoza J, Ruiz F. Synthesis and antibacterial activity of silver nanoparticles with different sizes. J Nanoparticle Res. 2008;10:1343–8. 10.1007/s11051-008-9428-6.

[26] Targino AGR, Flores MAP, dos Santos Junior VE, de Godoy BenéBezerra F, de Luna Freire H, Galembeck A, Rosenblatt A. An innovative approach to treating dental decay in children. A new anti-caries agent. J Mater Sci Mater Med. 2014;25:2041–7. 10.1007/s10856-014-5221-5. 24818873 10.1007/s10856-014-5221-5

[27] Aldhaian BA, Balhaddad AA, Alfaifi AA, Levon JA, Eckert GJ, Hara AT, Lippert F. In vitro demineralization prevention by fluoride and silver nanoparticles when applied to sound enamel and enamel caries-like lesions of varying severities. J Dent. 2021;104:103536. 10.1016/j.jdent.2020.103536. 33217487 10.1016/j.jdent.2020.103536

[28] Favaro JC, Detomini TR, Maia LP, Poli RC, Guiraldo RD, Lopes MB, Berger SB. Anticaries agent based on silver nanoparticles and fluoride: characterization and biological and remineralizing effects—an in vitro study. Int J Dent. 2022;2022. 10.1155/2022/9483589. 10.1155/2022/9483589 PMC9042602 35497178

[29] Petrie A, Sabin C. Medical statistics at a glance. 4th ed. London: Wiley-Blackwell; 2019.

[30] Dogan F, Civelek A, Oktay I. Effect of different fluoride concentrations on remineralization of demineralized enamel: An in vitro pH cycling study. OHDMBSC. 2004;1:20–6.

[31] Hasanin MT, Elfeky SA, Mohamed MB, Amin RM. Production of well-dispersed aqueous cross-linked chitosan-based nanomaterials as alternative antimicrobial approach. J Inorg Organomet Polym Mater. 2018;28:1502–10. 10.1007/s10904-018-0855-2.

[32] Loutfy SA, El-Din HMA, Elberry MH, Allam NG, Hasanin M, Abdellah AM. Synthesis, characterization and cytotoxic evaluation of chitosan nanoparticles: in vitro liver cancer model. Adv Nat Sci Nanosci Nanotechnol. 2016;7:035008. 10.1088/2043-6262/7/3/035008.

[33] Habib S, Sadek H, Hasanin M, Bayoumi R. Antimicrobial activity, physical properties and sealing ability of epoxy resin-based sealer impregnated with green tea extract-chitosan microcapsules: an in-vitro study. Egypt Dent J. 2021;67:2309–19. 10.21608/edj.2021.64866.1526.

[34] Jasem AJ, Mahmood MA. Preparation and characterization of amoxicillin-loaded chitosan nanoparticles to enhance antibacterial activity against dental decay pathogens. J Emerg Med Trauma Acute Care. 2023;2023:10. 10.5339/jemtac.2023.midc.10.

[35] Turkevich J, Stevenson PC, Hillier J. A study of the nucleation and growth processes in the synthesis of colloidal gold. Discuss Faraday Soc. 1951;11:55–75.

[36] Lee P, Meisel D. Adsorption and surface-enhanced Raman of dyes on silver and gold sols. J Phys Chem. 1982;86:3391–5. 10.1021/j100214a025.

[37] Noronha Mdos S, Romão DA, Cury JA, Tabchoury CP. Effect of fluoride concentration on reduction of enamel demineralization according to the cariogenic challenge. Braz Dent J. 2016;27:393–8. 10.1590/0103-6440201600831. 27652699 10.1590/0103-6440201600831

[38] Alsherif AA, Farag MA, Helal MB. Efficacy of nano silver fluoride and/or diode laser in enhancing enamel anticariogenicity around orthodontic brackets. BDJ Open. 2023;9:22. 10.1038/s41405-023-00151-x. 37353492 10.1038/s41405-023-00151-x PMC10290105

[39] Bezerra SJC, Viana ÍEL, Aoki IV, Duarte S, Hara AT, Scaramucci T. In-vitro evaluation of the anti-cariogenic effect of a hybrid coating associated with encapsulated sodium fluoride and stannous chloride in nanoclays on enamel. J Appl Oral Sci. 2022;30:e20210643. 10.1590/1678-7757-2021-0643. 35507984 10.1590/1678-7757-2021-0643 PMC9064272

[40] El-Naggar NEA, Shiha AM, Mahrous H, Mohammed AA. Green synthesis of chitosan nanoparticles, optimization, characterization and antibacterial efficacy against multi drug resistant biofilm-forming Acinetobacter baumannii. Sci Rep. 2022;12:19869. 10.1038/s41598-022-24303-5. 36400832 10.1038/s41598-022-24303-5 PMC9674591

[41] Khanmohammadi M, Elmizadeh H, Ghasemi K. Investigation of size and morphology of chitosan nanoparticles used in drug delivery system employing chemometric technique. IJPR. 2015;14:665. 26330855 PMC4518095

[42] Arnold WH, Bietau V, Renner PO, Gaengler P. Micromorphological and micronanalytical characterization of stagnating and progressing root caries lesions. Arch Oral Biol. 2007;52:591–7. 10.1016/j.archoralbio.2006.11.008. 17181998 10.1016/j.archoralbio.2006.11.008

[43] Bell DC, Garratt-Reed AJ. Energy dispersive X-ray analysis in the electron microscope: Garland Science; 2003. 10.4324/9780203483428.

[44] Gupta BD, Semwal V, Pathak A. Nanotechnology-based fiber-optic chemical and biosensors. In: Nano-optics: fundamentals, experimental methods, and applications. 2020. p. 163.

[45] Moore PL, Booth G. The welding engineer’s guide to fracture and fatigue. Cambridge: Elsevier Woodhead Publishing; 2015.

[46] Mohammed A, Abdullah A, editors. Scanning electron microscopy (SEM): A review. Proceedings of the 2018 International Conference on Hydraulics and Pneumatics—HERVEX, Băile Govora, Romania; 2018.

[47] De Carvalho M, Stamford TCM, Dos Santos E, Tenorio P, Sampaio F. Chitosan as an oral antimicrobial agent. Formatex. 2011;2012:13.

[48] Noaman KM, Al-Samoly WM, Al-Hariri AA-KH. Evaluation of the degree of remineralization of subclinical carious lesions using chitosan and conventional remineralizing agents (An in vitro study). Egypt J Hosp Med. 2020;78:182–9. 10.21608/EJHM.2020.69376.

[49] Li Y, Liu Y, Psoter WJ, Nguyen OM, Bromage TG, Walters MA, et al. Assessment of the silver penetration and distribution in carious lesions of deciduous teeth treated with silver diamine fluoride. Caries Res. 2019;53:431–40. 10.1159/000496210. 30808824 10.1159/000496210

[50] Scarpelli BB, Punhagui MF, Hoeppner MG, Almeida RSCd, Juliani FA, Guiraldo RD, Berger SB. In vitro evaluation of the remineralizing potential and antimicrobial activity of a cariostatic agent with silver nanoparticles. Braz Dent J. 2017;28:738–43. 10.1590/0103-6440201701365. 29211131 10.1590/0103-6440201701365

[51] Nozari A, Ajami S, Rafiei A, Niazi E. Impact of nano hydroxyapatite, nano silver fluoride and sodium fluoride varnish on primary teeth enamel remineralization: an in vitro study. JCDR. 2017;11:ZC97. 10.7860/JCDR/2017/30108.10694. 29207844 10.7860/JCDR/2017/30108.10694 PMC5713866

[52] Mei ML, Nudelman F, Marzec B, Walker J, Lo E, Walls A, Chu C-H. Formation of fluorohydroxyapatite with silver diamine fluoride. J Dent Res. 2017;96:1122–8. 10.1177/0022034517709738. 28521107 10.1177/0022034517709738 PMC5582683

[53] Freire PL, Albuquerque AJ, Farias IA, da Silva TG, Aguiar JS, Galembeck A, et al. Antimicrobial and cytotoxicity evaluation of colloidal chitosan–silver nanoparticles–fluoride nanocomposites. Int J Biol Macromol. 2016;93:896–903. 10.1016/j.ijbiomac.2016.09.052. 27642129 10.1016/j.ijbiomac.2016.09.052

[54] dos Santos Jr VE, VasconcelosFilho A, Targino AGR, Flores MAP, Galembeck A, Caldas AF Jr, Rosenblatt A. A new “silver-bullet” to treat caries in children–nano silver fluoride: a randomised clinical trial. J Dent. 2014;42:945–51. 10.1016/j.jdent.2014.05.017. 24930870 10.1016/j.jdent.2014.05.017

[55] Soares R, De Ataide IDN, Fernandes M, Lambor R. Assessment of enamel remineralisation after treatment with four different remineralising agents: a scanning electron microscopy (SEM) study. J Clin Diagn Res. 2017;11:ZC136. 10.7860/JCDR/2017/23594.9758. 28571281 10.7860/JCDR/2017/23594.9758 PMC5449906

[56] Wang H, Xiao Z, Yang J, Lu D, Kishen A, Li Y, et al. Oriented and ordered biomimetic remineralization of the surface of demineralized dental enamel using HAP@ ACP nanoparticles guided by glycine. Sci Rep. 2017;7:40701. 10.1038/srep40701. 28079165 10.1038/srep40701 PMC5228061

[57] Salvati E, Besnard C, Harper RA, Moxham T, Shelton RM, Landini G, Korsunsky AM. Finite element modelling and experimental validation of the enamel demineralisation process at the rod level. J Adv Res. 2021;29:167–77. 10.1016/j.jare.2020.08.018. 33842014 10.1016/j.jare.2020.08.018 PMC8020348

[58] Besnard C, Harper RA, Salvati E, Moxham TE, Brandt LR, Landini G, et al. Analysis of in vitro demineralised human enamel using multi-scale correlative optical and scanning electron microscopy, and high-resolution synchrotron wide-angle X-ray scattering. Mater Des. 2021;206:109739. 10.1016/j.matdes.2021.109739.

[59] Mei ML, Lo EC, Chu C. Arresting dentine caries with silver diamine fluoride: what’s behind it? J Dent Res. 2018;97:751–8. 10.1177/0022034518774783. 29768975 10.1177/0022034518774783

[60] Zhi Q, Lo E, Kwok A. An in vitro study of silver and fluoride ions on remineralization of demineralized enamel and dentine. Aust Dent J. 2013;58:50–6. 10.1111/adj.12033. 23441792 10.1111/adj.12033

[61] Hernández-Sierra JF, Ruiz F, Pena DCC, Martínez-Gutiérrez F, Martínez AE, Guillén AdJP, et al. The antimicrobial sensitivity of Streptococcus mutans to nanoparticles of silver, zinc oxide, and gold. NBM. 2008;4:237–40. 10.1016/j.nano.2008.04.005. 10.1016/j.nano.2008.04.005 18565800

[62] Zhang K, Cheng L, Imazato S, Antonucci JM, Lin NJ, Lin-Gibson S, et al. Effects of dual antibacterial agents MDPB and nano-silver in primer on microcosm biofilm, cytotoxicity and dentine bond properties. J Dent. 2013;41:464–74. 10.1016/j.jdent.2013.02.001. 23402889 10.1016/j.jdent.2013.02.001 PMC3654025

